# Young gut microbiota transplantation improves the metabolic health of old mice

**DOI:** 10.1128/msystems.01601-24

**Published:** 2025-05-30

**Authors:** Jiaojiao Xie, Taewan Kim, Zhongmao Liu, Hunter Panier, Suresh Bokoliya, Ming Xu, Yanjiao Zhou

**Affiliations:** 1 The First Affiliated Hospital, Zhejiang University School of Medicine 71069, Hangzhou, Zhejiang, China; 2 Department of Medicine, University of Connecticut Health Center 396717, Farmington, Connecticut, USA; 3 Center of Aging, University of Connecticut Health Center 396717, Farmington, Connecticut, USA; Cleveland Clinic, Cleveland, Ohio, USA

**Keywords:** fecal microbiota transplantation, aging, depression, anxiety, gut microbiota, lipid, amino acids

## Abstract

The gut microbiota evolves over a lifetime and significantly impacts the aging process. Targeting the gut microbiota represents a novel avenue to delay aging and aging-related physical and mental decline. However, the underlying mechanism by which the microbiota modulates the aging process, particularly age-related physical and behavioral changes is not completely understood. We conducted fecal microbiota transplantation (FMT) from young or old male donor mice to the old male recipients. Old recipients with young microbiota had a higher alpha diversity than the old recipients with old microbiota. Compared to FMT with old microbiota, FMT with young microbiota reduced body weight and prevented fat accumulation in the old recipients. FMT with young microbiota also lowered frailty, increased grip strength, and alleviated depression and anxiety-like behavior in the old recipients. Consistent with observed physical changes, untargeted metabolomic analysis of serum and stools revealed that FMT with young microbiota lowered age-related long-chain fatty acid levels and increased amino acid levels in the old recipients. Bulk RNAseq analysis of the amygdala of the brain showed that FMT with young microbiota downregulated inflammatory pathways and upregulated oxidative phosphorylation in the old recipients. Our results demonstrate that FMT with young microbiota has substantial positive influences on age-related body composition, frailty, and psychological behaviors. These effects are associated with changes in host lipid and amino acid metabolism in the periphery and transcriptional regulation of neuroinflammation and energy utilization in the brain.

**IMPORTANCE:**

The gut microbiome is a key hallmark of aging. Fecal microbiota transplantation (FMT) using young microbiota represents a novel rejuvenation strategy to delay aging. Our study provides compelling evidence that transplanting microbiota from young mice significantly improved grip strength, frailty, and body composition in aged recipient mice. At the molecular level, FMT improved aging-related metabolic markers in the gut and circulation. Additionally, FMT from young microbiota rejuvenated the amygdala of the aged brain by downregulating inflammatory pathways. This study highlights the importance of metabolic reprogramming via young microbiota FMT in improving physical and metabolic health in elderly recipients.

## INTRODUCTION

Aging is a gradual process of deterioration of physical and mental functions, which is demonstrated by impaired metabolic, immune, and cognitive functioning at a systemic level. The gut microbiota, comprised of trillions of microorganisms along the gastrointestinal tract, is an essential player in the modulation of host metabolism, immune responses, and gut–brain communication (1). The changing gut microbiota from infancy through adulthood and into later life provides a new perspective for understanding the biology of aging and identifying innovative strategies to delay aging and ameliorate aging-related diseases.

The gut microbiome is emerging as a new hallmark of aging (2). Studies have shown patterned changes in the gut microbiome in aging, including decreased beta diversity and increased relative abundance of pathogenic microbes (3, 4). In addition, fecal microbiota transplantation (FMT) that transfers the gut microbiota from old donors to young recipients or vice versa provides direct evidence that the gut microbiota can modify age-related pathophysiology. FMT from the older donors to the young recipients decreased life span in fly and fish models of aging (5), impaired cognition behavior in rats (6), and reduced spatial learning and memory in mice (7). The old microbiota also shows obesogenic characteristics, leading to a higher fat body mass and higher blood insulin levels in young mice that received microbiota from aged donors than younger donors (8). While most studies have shown the detrimental effect of microbiota derived from old donors (old microbiota) on young animals, one study revealed that old microbiota induced neurogenesis and activated longevity signaling in young germ-free mice (9). Conversely, in the opposite direction, FMT from young donors to old recipients rejuvenated blood stem cells (10), extended health span, and life span in progeroid mice (11). Microbiota derived from young donors (young microbiota) rejuvenated the aged brain by restoring aging-associated neurocognitive impairments (12–15). However, investigation into the effects of young microbiota on neurophysiological behavior is still in its early stages. Only a few studies have been conducted with inconsistent findings on specific cognitive behavior tests.

In addition, the mechanisms underlying FMT-induced physiological changes in aging are not well understood. One fundamental function of the gut microbiota is to regulate host metabolism, including digestion, absorption, and storage of macronutrients, micronutrients, and xenobiosis, through which numerous diets or microbiome-derived metabolites are generated in the gut, enter circulation, and subsequently modulate a plethora of host functions (16, 17). Recent studies have found that 15% of blood metabolites are directly or indirectly attributed to the metabolism of the gut microbiota (18). Blood metabolites play an essential role in modulating the aging process. Numerous blood metabolic biomarkers have been linked to longevity, cognition, and psychological conditions in the aged population (19, 20). Young blood rejuvenates tissues and reverses the signs of aging in old mice (21). However, changes in gut and blood metabolites in older mice that received FMT from young donors, as well as their associations with physical function and psychological behaviors, have not been investigated.

The goal of this study is to determine the impact of FMT with young microbiota on the physical function and psychological behaviors in old recipients and to explore potential metabolic and transcriptomic mechanisms underlying these changes. Compared to FMT with old microbiota, we found that FMT with young microbiota reduced body mass, improved fragility, increased grip strength, and ameliorated depression and anxiety-like behavior in old recipient mice. These changes were accompanied by a significant reduction of long-chain fatty acids, an increase in amino acid metabolism in the serum, and changes in pathways involving neuroinflammation and energy utilization in the brain.

## MATERIALS AND METHODS

### Animal and study design

Young male adult C57BL/6J mice (3 months old, obtained from the Jackson Laboratory) and old male C57BL/6 mice (20 months old, obtained from National Institute on Aging [NIA]) were used in the study. Mice were fed Teklad Global 18% protein rodent diet (2918, Inotiv, WI, USA) and maintained in standard housing conditions throughout the entire duration of the study. The study included three groups (15 mice/group): FMT from old donors to old recipients (OO group), FMT from young donors to old recipients (YO group), and FMT from young donors to young recipients (YY group). At baseline, assessments of physical function (including frailty and grip strength), body composition (utilizing total dissolved nuclear magnetic resonance [TD-NMR]), body weight, and stool collection were conducted as detailed below. Then, mice were subjected to a 5-day course of oral antibiotic treatment and subsequent FMT, administered twice weekly until the end of the experiment. In addition to physical function and body composition measurements, open-field tests, elevated zero maze, tail suspension tests, and comprehensive laboratory animal monitoring system (CLAMS) assessments were also performed after 2 months of FMT. Mice were euthanized upon detection of significant changes in the open-field test data. Fecal pellets, ileum content, and colon content were collected for the microbiome analysis. Cecal content and serum were collected for the metabolomic analysis. The amygdala was harvested for transcriptomic analysis.

### Physical function measurement

The physical performance of each group was assessed by grip strength and frailty index. Grip Strength Test Meter (Bioseb, Pinellas Park, FL) was used to test the forelimb grip strength. Each mouse was tested with 10 trials, and average measurements were used for analysis. Thirty-one frailty index items were used to measure the frailty test as described previously (22), including integument, physical/musculoskeletal, vestibulocochlear/auditory, digestive/urogenital, respiratory, discomfort, and other systems, and the average frailty index score was calculated for each mouse. Based on the severity of each deficit, each mouse was scored either 1, 0.5, or 0. The Wilcoxon rank-sum test was performed to compare group differences at baseline and at the end of the study and to determine whether changes in physical function measurement were different from 0. All *P*-values were adjusted for multiple comparisons using a false discovery rate (FDR). An adjusted *P*-value of less than 0.1 or 0.05 was reported. The same statistical analysis was performed for body composition measurement, CLAMS measurements, and behavioral tests.

### Body composition measurement

Body composition measurements of mice were determined using a minispec mq7.5 TD-NMR analyzer (the ‘‘LF50,’’ Bruker, Billerica, MA). Fat or lean mass was measured and analyzed using the Minispec plus software v.7.0 (Bruker).

### CLAMS

The daily activity and food intake of each mouse were quantified using the CLAMS instrument. Mice were individually housed in chambers under a 12 h light/dark cycle, with free access to water and food for 3 consecutive days. Activity, food consumption, and water intake were monitored and recorded by Oxymax-CLAMS software v.5.51. Data were further analyzed with the CLAMS Examination Tool (CLAX) v.2.2.15.

### Behavioral tests

#### Open field

The open-field test is useful when studying central and peripheral pathways involved in locomotion, anxiety, and exploration. An apparatus (45 × 45 × 45 cm, length × width × height) was centered under the camera and aligned into 16 squares (four corner zones and four center zones). A mouse was placed in the apparatus for 10 min. Total distance, average speed, number of entries to each zone, time in the zone, and distance traveled in the zone were recorded by an overhead camera and analyzed using an automated video tracking system. Between tests, the apparatus was cleaned with 70% ethanol.

#### Elevated zero maze

The elevated zero maze was an additional test used to measure anxiety. Briefly, a round apparatus (60 cm in diameter × 100 cm height) was centered under the camera and aligned into four areas (two open zones with 15 cm walls and two closed zones with 1 cm walls). A mouse was allowed to explore the maze for 5 min. An overhead camera and Any-Maze software were used to track the position of the mice and calculate the time spent in the open zones of the mazes and the distance traveled. Between tests, the apparatus was cleaned with 70% ethanol.

#### Tail suspension test

The tail suspension test is a common test to measure depression. Mice are suspended by their tails above the floor at 25 cm with tape for 6 min and recorded with a camera. Four mice were tested at a time. Escape-oriented behaviors, including trying to reach the walls of the apparatus and the suspension bar, strong shaking of the body, and movement of the limbs akin to running, are quantified. Between tests, the apparatus was cleaned with 70% ethanol.

### FMT

To prepare for donor stools, stool pellets were collected and pooled from 15 young or 30 old mice for 1 week. Pooled fecal pellets were weighed, vortexed, and dissolved in reduced brain heart infusion (BHI) (20 mg/200 µL), followed by a quick spin to remove large debris. The resulting fecal liquid was aliquoted and stored at −80℃ until FMT. To prepare for recipients, the same donor mice were pretreated with an oral antibiotic cocktail consisting of vancomycin (0.5 g/L), neomycin (1 g/L), ampicillin (1 g/L), and metronidazole (1 g/L) once a day for 5 days, followed by FMT through oral gavage of 200 µL fecal liquid/mouse twice a week for 2 months.

### 16S rRNA gene sequencing and data analysis

Fecal pellets, ileum content, and colon content samples (*n* = 12–14 mice/group) were collected and frozen at −80°C. DNA extraction was performed with a DNeasy PowerSoil Pro Kit (QIAGEN) according to the manufacturer’s instructions. The V4 hypervariable region of 16S rRNA was PCR amplified. The PCR amplicon was sequenced at the Illumina MiSeq platform, targeting 10,000 reads/sample. The raw reads from each sample were processed through the DADA2 package (v.3.6.3). Principal component analysis (PCA) was conducted to view microbiome distribution patterns at the genus level using the MicroViz package. Alpha diversity, including Chao1, Shannon, and Simpson indices, was calculated using the Phyloseq package. The between-group difference of alpha diversity was compared by the Wilcoxon rank-sum test. Linear discriminant analysis (LDA) effect size (LefSe) was used to identify differential taxa between groups. *P*-values were adjusted from multiple comparisons. An adjusted *P*-value of less than 0.1 was considered statistically significant.

### Transcriptomics for amygdala

Total RNA of the amygdala (*n* = 5 mice/group) was extracted using TRIzol reagent (Invitrogen, Thermo Fisher Scientific), purified with chloroform, isopropanol, and 75% ice-cold ethanol (Sigma-Aldrich), and dissolved in RNase-free water. Extracted RNAs were sent to the UConn Computational Biology Core to perform RNA sequencing. Raw reads were subjected to adaptor removal and filtering of low-quality reads. Filtered reads were mapped to the mouse reference genome using STAR (Spliced Transcripts Alignment to a Reference). The gene expression level was estimated by transcripts per million of transcript sequences. Gene set enrichment analysis (GSEA) was performed using GSEA software v.4.3.2 (23). The hallmark gene set database provided by the software was used to assess significantly enriched hallmark pathways. Enriched pathways with *P*-value <0.05 and FDR <0.25 were considered statistically significant.

### Metabolomics for stool and serum

Serum and stool samples in YO and OO groups (*n* = 4 or 5 mice/group) were collected at the end of the study and sent to Metabolon Inc. for untargeted metabolome analysis using ultrahigh performance liquid chromatography-tandem mass spectroscopy platform. Peaks were quantified using area under the curve. Following normalization to volume for serum samples, log transformation, and imputation of missing values, if any, with the minimum observed value for each metabolite, analysis of variance contrasts and Welch’s two-sample *t*-test were used to identify metabolites that differed significantly between the OO and YO groups. An estimate of the false discovery rate is calculated to take into account the multiple comparisons. An adjusted *P*-value of less than 0.1 was considered statistically significant. Metabolites with statistical differences were plotted by heatmap along with hierarchical clustering. Euclidean distance and complete linkage were used to construct the clustering using the hclust package in R.

## RESULTS

### FMT with young microbiota lowers body weight, improves physical function, and attenuates anxiety and depression-like behavior in the old recipients

**Fig 1:**
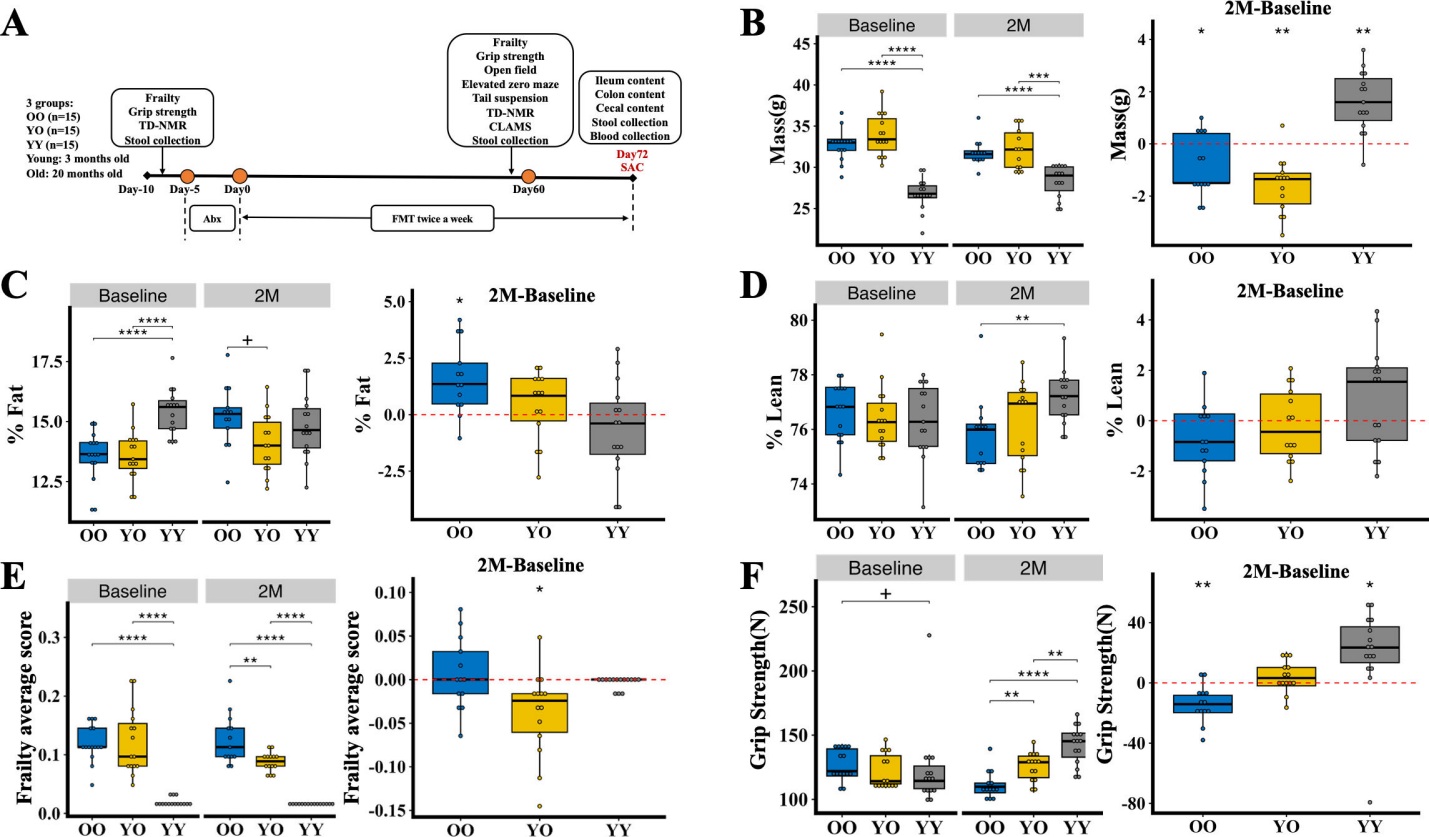
Body composition and physical function in OO, YO, and YY groups before and after FMT. Overview of the study design and timeline. Three groups were included: OO (old-to-old FMT), YO (young-to-old FMT), and YY (young-to-young FMT), with 15 male mice per group. Old mice were 20 months old and young mice were 3 months old. Physical functions such as frailty and grip strength were conducted at baseline and after 2 months of FMT. Behavioral tests, including open-field, elevated zero maze, and tail suspension tests, were tested after 2 months of FMT. “Abx” refers to antibiotic treatment (**A**). Between-group comparisons of body weight (**B**), percentage of fat (**C**), percentage of lean mass (**D**), frailty score averaged from 31 indexes (**E**), and grip strength (**F**) at baseline and after 2 months of FMT. Within-group comparisons of changes in body weight (**B**), percentage of fat (**C**), percentage of lean mass (**D**), frailty average score (**E**), and grip strength (**F**) from baseline to 2 months of FMT (2M-Baseline). All comparisons are done by Wilcoxon tests, and *P*-values are adjusted for multiple comparisons using FDR. For adjusted *P*-values: ^+^*P* < 0.1, *P < 0.05, **P < 0.01, ***P < 0.001, and ****P < 0.0001.

To determine the effects of young microbiota on the physical and psychological behaviors of the old recipients, we performed FMT twice a week for 2 months in three groups of mice (*n* = 15 mice/group), including YO group, OO group, and YY group. In the YO group, FMT was performed by transplantation of stools of young donors (3 months old mice) to old recipients (20 months old mice), while in the OO group and YY group, FMT was performed by transplantation of stools of old donors (20 months old mice) to old recipients (20 months old mice) and young donor to young recipients, respectively (Fig. 1A). To first determine whether FMT with young microbiota affects body weight and body composition of old recipients, we compared body weight and percentage of fat and percentage of lean mass in three groups at baseline and at 2 months after FMT, as well as changes in body weight and percentage of fat and lean mass from baseline to 2 months in each group.

At baseline before FMT, older mice exhibited significantly higher body weight and lower percentage of fat compared to their younger counterparts, but no differences were observed between the OO and YO groups (Fig. 1B and C, baseline). The percentage of lean mass was the same in the young and old mice (Fig. 1D, baseline). After 2 months of FMT, body weight differences between groups remained the same as baseline (Fig. 1B, 2M). Within-group comparison of changes in body weight from baseline to 2 months showed that body weight decreased significantly in the OO and YO groups, but the degree of decrease was much higher in the YO group. By contrast, body weight increased in the YY group from baseline to 2 months (Fig. 1B, 2M-Baseline). After 2 months of FMT, the percentage of fat in the YO group was lower than in the OO group (Fig. 1C, 2M). Within-group comparison revealed that the OO group had a significant increase in the percentage of fat (Fig. 1C, 2M-Baseline), and the YO group had no statistical changes. In terms of percentage of lean body mass, after 2 months of FMT, the OO group had significantly lower levels of lean body weight than the YY group, whereas no statistical differences were observed in the YO group compared to other groups (Fig. 1D, 2M). The percentage of lean showed no significant changes in all three groups (Fig. 1D, 2M-Baseline). In addition, food intake in the YO and OO groups was similar (Fig. S1B). These data indicate that FMT with young microbiota can reduce body weight and prevent fat accumulation in old recipients.

**Fig 2:**
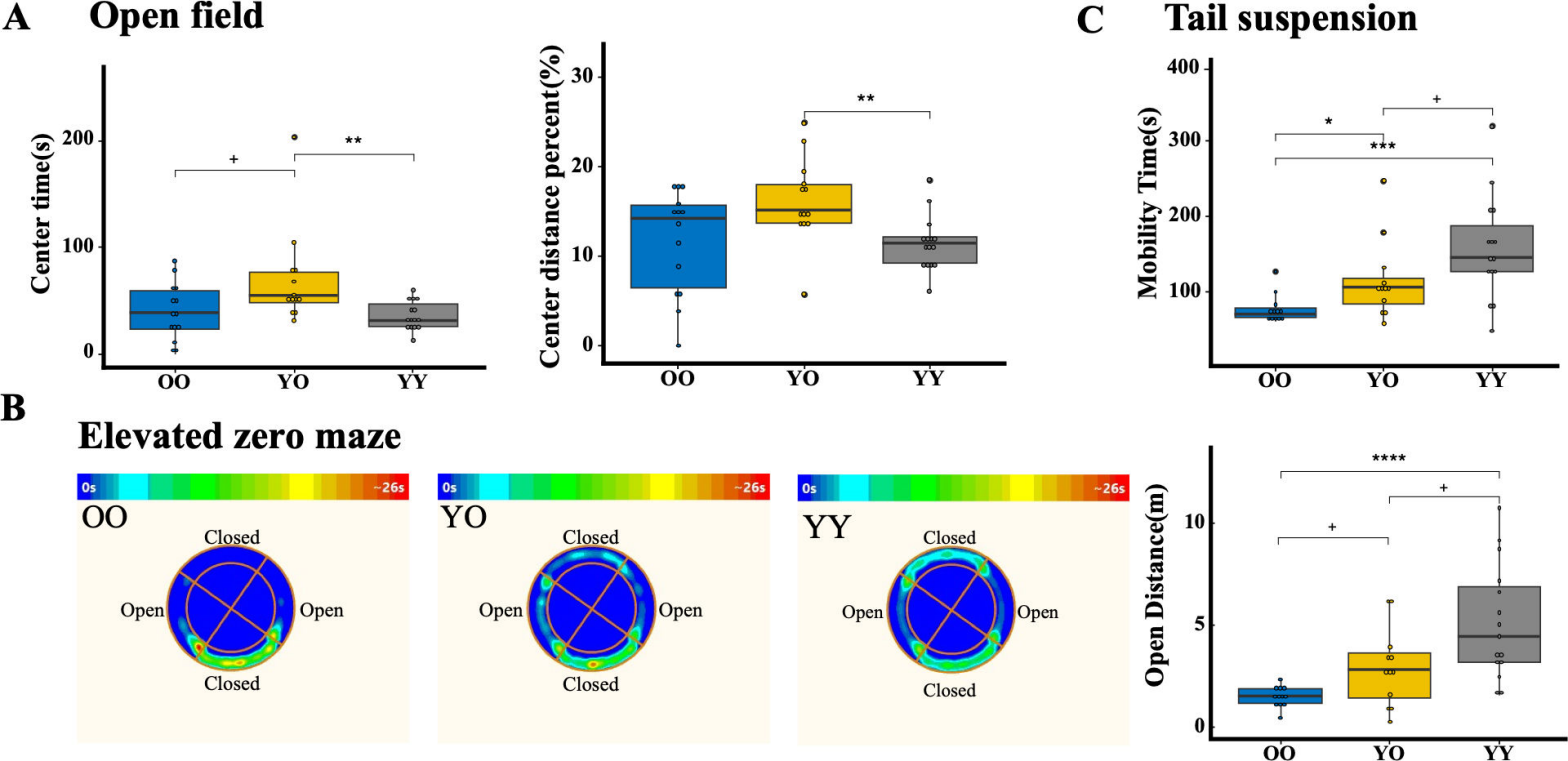
Behavioral tests in OO, YO, and YY groups after 2 months of FMT. (**A**) Open-field tests. YO mice spend more time and travel more distance in the center area compared to the OO group and YY group. (**B**) Elevated zero-maze tests. YO mice spend more time and travel more distance in the open area compared to the OO group. (**C**) Tail suspension tests. YO mice have longer mobility time than OO mice. *P < 0.05, **P < 0.01, ***P < 0.001, ****P < 0.0001.

We next examined the physical functions such as frailty score and grip strength in the three groups. As expected, at baseline, the OO and YO groups were frailer than the YY group, as shown by significantly higher frailty scores in the OO and YO groups (Fig. 1E, Baseline). After FMT, the between-group comparison showed that the average frailty score became significantly lower in the YO group than in the OO group (Fig. 1E, 2M). Within-group comparison also revealed a decreased average frailty score in the YO group before and after FMT (Fig. 1E, 2M-Baseline). At baseline, grip strength was different between the OO and YY groups. However, FMT led to significantly higher levels of grip strength in the YO group than in the OO group (Fig. 1F, 2M). Within-group comparison before and after FMT showed the grip strength in the OO group decreased significantly while the YO group maintained a similar level (Fig. 1F, 2M-Baseline). These data suggest that FMT with young microbiota improves frailty and prevents deterioration of grip strength in the old recipients.

To assess how FMT with young microbiota affects anxiety and depression-like behaviors, we performed an open-field test, elevated zero-maze test, and tail suspension test at the end of the FMT. The YO group spent more time in the center sections than the OO group and the YY group. The YO group also had a significantly higher percentage of travel distance in the center sections than the YY group (Fig. 2A). As depicted in Fig. 2B of the elevated zero-maze test (heatmap image), the YO group tended to travel to another part of the area from the experiment starting point, which contrasted with the OO group that preferred to stay in the closed area of the experiment starting point. Statistically, the YO group traveled more distance in the open area than the OO group and less than the YY group (Fig. 2B). The YO group showed increased mobility time in the tail suspension test compared to the OO (Fig. 2C). These results strongly suggest a potential beneficial impact of FMT with young microbiota in alleviating anxiety and depression-like phenotypes.

### FMT with young microbiota reshapes the microbial community of old recipients

**Fig 3:**
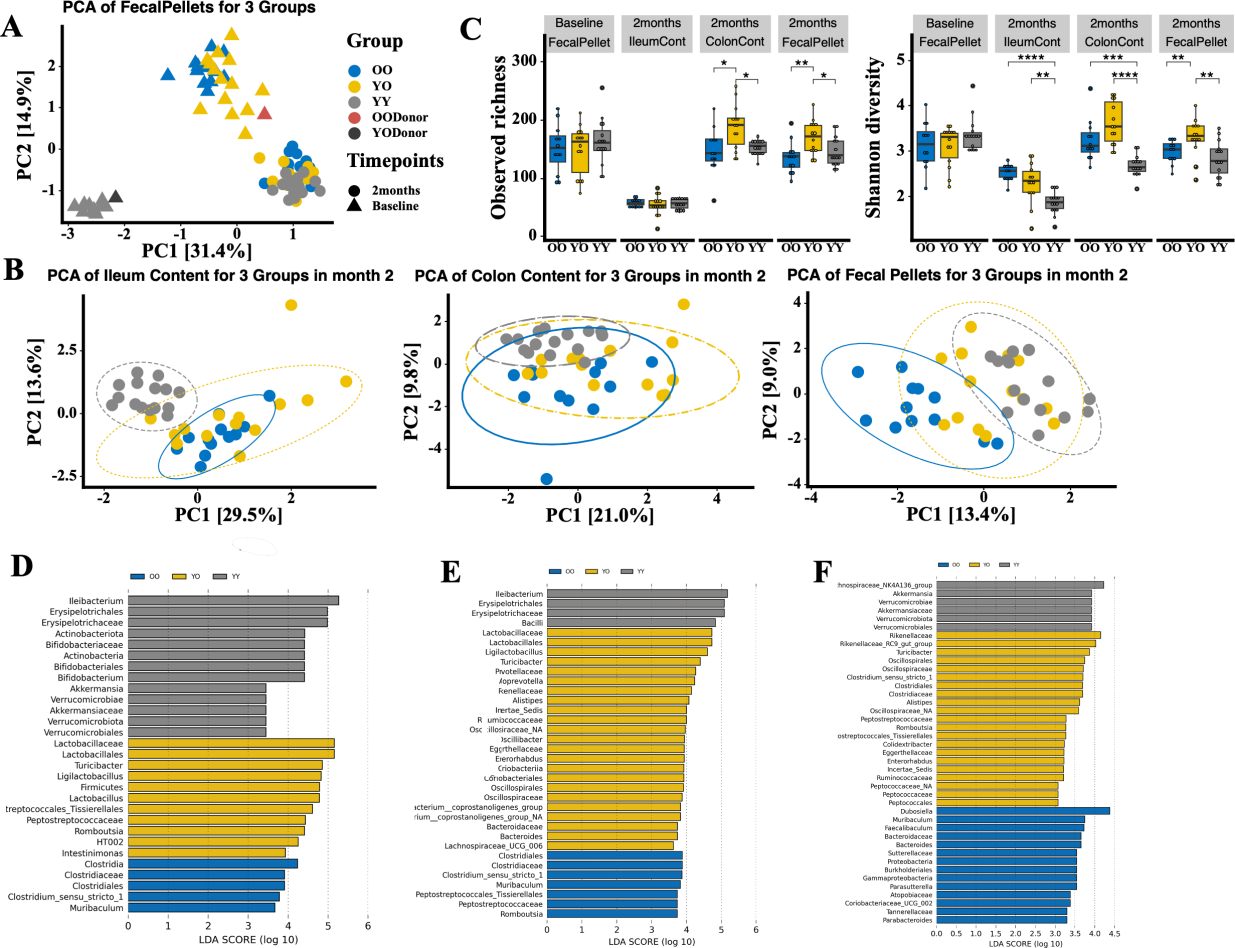
The gut microbiome in OO, YO, and YY groups before and after FMT. (**A**) The PCA of fecal microbiome in the three groups before and after FMT. (**B**) PCAs of microbiome from ileum content, colon content, and fecal pellets after FMT. The area of an ellipse contains 95% of samples for each group. (**C**) Observed richness and Shannon diversity of the microbiome in ileum content, colon content, and fecal pellets before and after FMT. (**D–F**) Bar charts showing the log-transformed LDA scores of differential bacterial taxa enriched in the three groups in ileum content (**D**), colon content (**E**), and fecal pellets (**F**) by LefSe analysis. Taxa with *P* < 0.05 and log-transformed LDA score >2 are plotted.

To determine how FMT changes the microbiota in recipient mice, we performed 16S rRNA gene sequencing of donor stools and recipient stools at baseline and stool, ileum, and colon contents after FMT at the end of the study. PCA, including both baseline and endpoint data, showed a distinct separation between young and old microbiota at baseline, and FMT significantly shifted the microbiota in both young and old recipients (Fig. 3A). PCA of the ileum, colon contents, and fecal pellets of three groups after FMT indicated that the YO group formed its own community structure that was different from both the OO and YY groups (Fig. 3B). The microbial alpha diversity was significantly higher in the YO group than in the OO and YY groups after FMT, which was evident in the ileum, colon, and fecal samples (Fig. 3C). We further identified specific microbiota differences after FMT in three groups using the LefSe analysis. As microbiome communities vary across different segments of the gastrointestinal tract, differential microbiota identified in the ileum (Fig. 3D), colon (Fig. 3E), and stools (Fig. 3F) of the three groups were not completely the same. *Akkermansia*, a genus that has shown anti-aging effects, was enriched in the ileum and stool of the YY group. Bacteria from the *Clostridia* class were enriched in the ileum and colon of the OO group. The OO group was also seen to increase pro-inflammatory bacteria such as *Gammaproteobacteria*. Interestingly, *Turicibacter* was enriched in the YO group across the ileum, colon, and stool samples, and *Lactobacillus* was enriched in the YO group in both the ileum and colon. *Lactobacillus* is a well-known probiotic-like bacteria with multiple health benefits, and *Turicibacter* has been shown to improve lipid metabolism (24) and possible anti-inflammatory effects (25). Together, our data suggest that FMT with young microbiota reshapes the microbiome community of old recipients, and it may preferentially promote the growth of beneficial taxa.

### Impact of FMT with young microbiota on gut metabolites in old recipients

**Fig 4:**
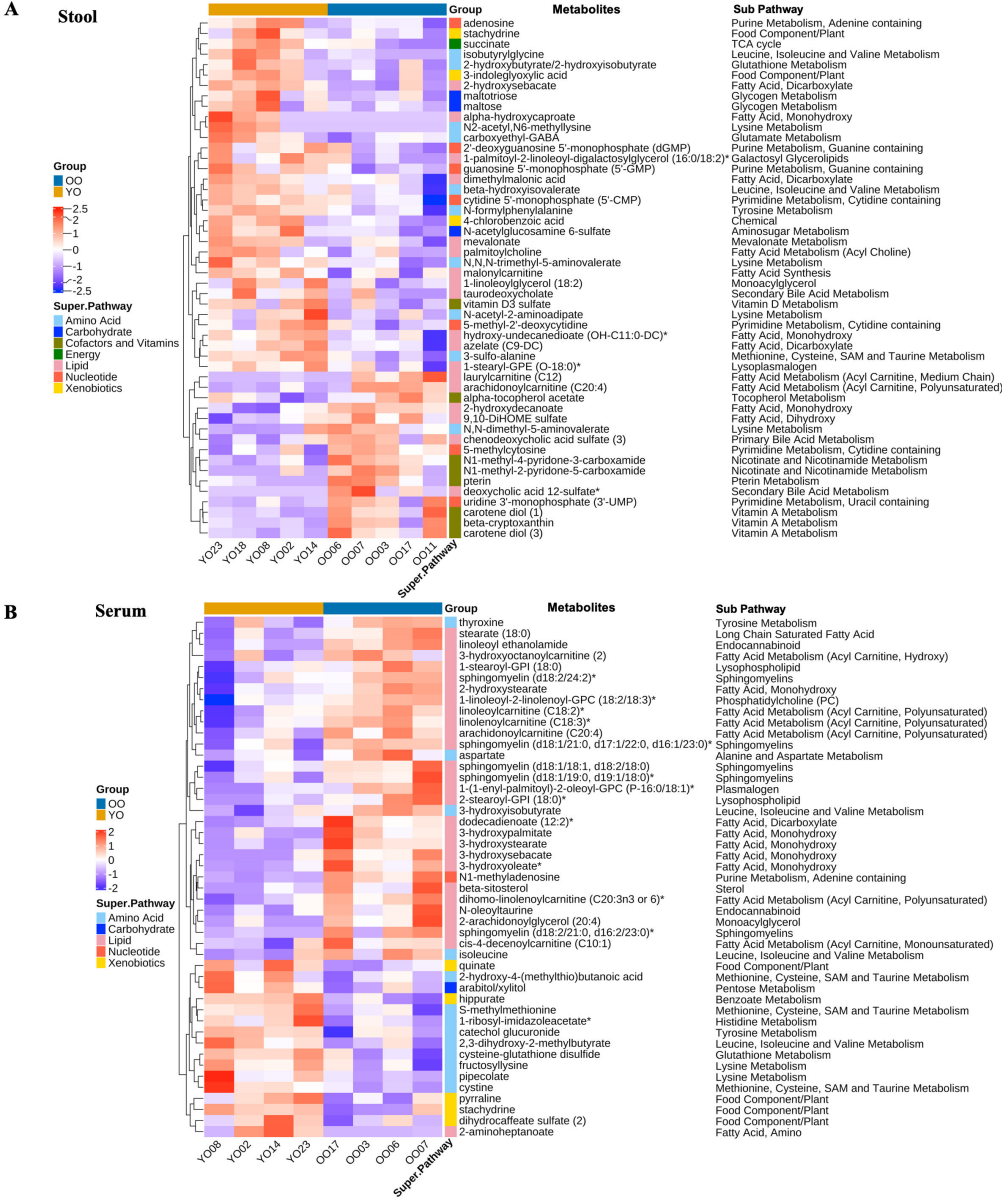
Differential stool and serum metabolites in the OO and YO groups after FMT. (**A**) The heatmap of stool metabolites that are significantly different between the YO and OO groups. (**B**) The heatmap of serum metabolites that are significantly different between the YO and OO groups. Metabolites in stool and serum were identified by untargeted metabolomics and compared between the OO and YO groups by the Welch’s *t*-test. The potential subpathway involving the metabolites is listed on the right of the metabolites. Superpathways are indicated in different colors. The density of the color in each cell of the heatmap represents the Z-score transformation of metabolite values.

To explore the possibility that FMT with young microbiota alters physical functions and behaviors in the old recipients through modulation of metabolites, we performed untargeted metabolome analysis in both stool and serum samples in the YO and OO groups. In total, 1,064 gut metabolites were identified. Fifty metabolites (18 related to lipids, 10 amino acids, 8 cofactors and vitamins, 7 nucleotides, 3 carbohydrates, 3 xenobiotics, and 1 energy superpathway) were significantly different between YO and OO groups in stool samples (Fig. 4A). Regarding lipid-related metabolites, the YO group had significantly lower levels of acyl carnitine, medium-chain (laurylcarnitine [C12]) and polyunsaturated (arachidonoylcarnitine [C20:4]) fatty acid than the OO group. Levels of 9,10-DiHOME sulfate—a leukotoxin derivative of linoleic acid diol—as well as sulfated primary bile acids (such as chenodeoxycholic acid sulfate) and secondary bile acids (like deoxycholic acid 12-sulfate) were reduced in the YO group compared to the OO group. In converse, we found that 12 gut metabolites related to lipid metabolism were significantly enriched in the YO group. The functionality for the majority of them is unknown except for conjugated secondary bile acid taurodeoxycholate and azelate (C9-DC), both of which have been shown to have potent anti-inflammatory properties (26, 27).

Interestingly, 9 out of 10 metabolites related to amino acid metabolism were upregulated in the YO group. These included 2-hydroxybutyrate/2-hydroxyisobutyrate involved in glutathione metabolism, 3-sulfo-alanine involved in methionine, cysteine, S-adenosylmethionine (SAM), and taurine metabolism, beta-hydroxyisovalerate and isobutyrylglycine involved in leucine, isoleucine, and valine metabolism, and several metabolites involved in tyrosine and lysine metabolism. The results suggest that FMT with young microbiota may upregulate amino acid metabolism in the gut of the old recipients.

We also observed the upregulation of three metabolites related to carbohydrate metabolism, including maltose, maltotriose, and N-acetylglucosamine 6-sulfate. Upregulation of metabolism was also observed in purine and pyrimidine metabolism and tricarboxylic acid cycle (TCA) cycles. Recent studies suggest that the gut microbiome regulates xenobiotic metabolism (28). We found FMT with young microbiota increased three metabolites (3-indoleglyoxylic acid, 4-chlorobenzoic acid, and stachydrine) responsible for xenobiotic metabolism in the YO group.

Seven metabolites involved in the metabolism of vitamins A, B, and E (alpha-tocopherol acetate) were all decreased in the YO group. By contrast, vitamin D3 sulfate was increased in the YO group.

Overall, our data suggest that FMT with young microbiota impacts metabolism in the gut of old recipients by upregulating amino acid, carbohydrate, nucleotide, and xenobiotic metabolisms and by downregulating vitamin A, B, and E metabolisms in general. FMT with young microbiota also upregulates or downregulates gut lipid metabolism depending on specific metabolites.

### Impact of FMT with young microbiota on serum metabolites in old recipients

In total, 946 serum metabolites were identified by untargeted serum metabolites. Forty-seven metabolites were significantly different between the YO and OO groups, with 27 metabolites related to lipid metabolism, 13 related to amino acid metabolism, 5 related to xenobiotic metabolism, 1 related to nucleotide, and 1 related to carbohydrate metabolism (Fig. 4B). Strikingly, all the differential lipid metabolites were reduced in the YO group compared to the OO group. These included several polyunsaturated acyl carnitine (middle- and long-chain fatty acids), several monohydroxy fatty acids, long-chain saturated fatty acid stearate (18:0), lysophospholipid 1-stearoyl-glycosylphosphatidylinositol (GPI) (18:0) and 2-stearoyl-GPI (18:0), several sphingomyelins, two metabolites from endocannabinoid metabolism, monoacylglycerol 2-arachidonoylglycerol (20:4), and one metabolite from phosphatidylcholine and one from plasmalogen. Notably, we found polyunsaturated acyl carnitine arachidonoylcarnitine (C20:4) and long-chain saturated fatty acid stearate (18:0) were concomitantly decreased in gut and serum metabolites in the YO group.

Most differential amino acids were significantly increased in the YO group, including cysteine-glutathione disulfide involved in glutathione metabolism, cystine, and S-methylmethionine, and fructosyllysine and pipecolate involved in lysine metabolism. The upregulation of amino acid metabolism observed in the serum was consistent with metabolic changes observed in the gut of the YO group. However, 3-hydroxybutyrate—a metabolite of the BCAA valine—along with another BCAA, isoleucine, as well as aspartate and thyroxine, were decreased in the YO group.

As observed in xenobiotic metabolism in the gut, we found increased xenobiotic metabolism in the YO group. Increased metabolites include hippurate—a metabolite from food preservative sodium benzoate, and a metabolite from food components/plants such as stachydrine. Stachydrine level was increased in both the gut and serum of the YO group.

In summary, FMT with young microbiota has a significant impact on lipid, amino acid, and xenobiotic metabolism in the old recipient. FMT with young microbiota decreases a wide range of lipid metabolites in both stool and serum, particularly polyunsaturated acyl carnitine and saturated long-chain fatty acids, while increasing metabolites associated with amino acid and xenobiotic metabolism in old recipient mice.

### FMT with young microbiota alters transcriptomics in the amygdala in old recipients

**Fig 5:**
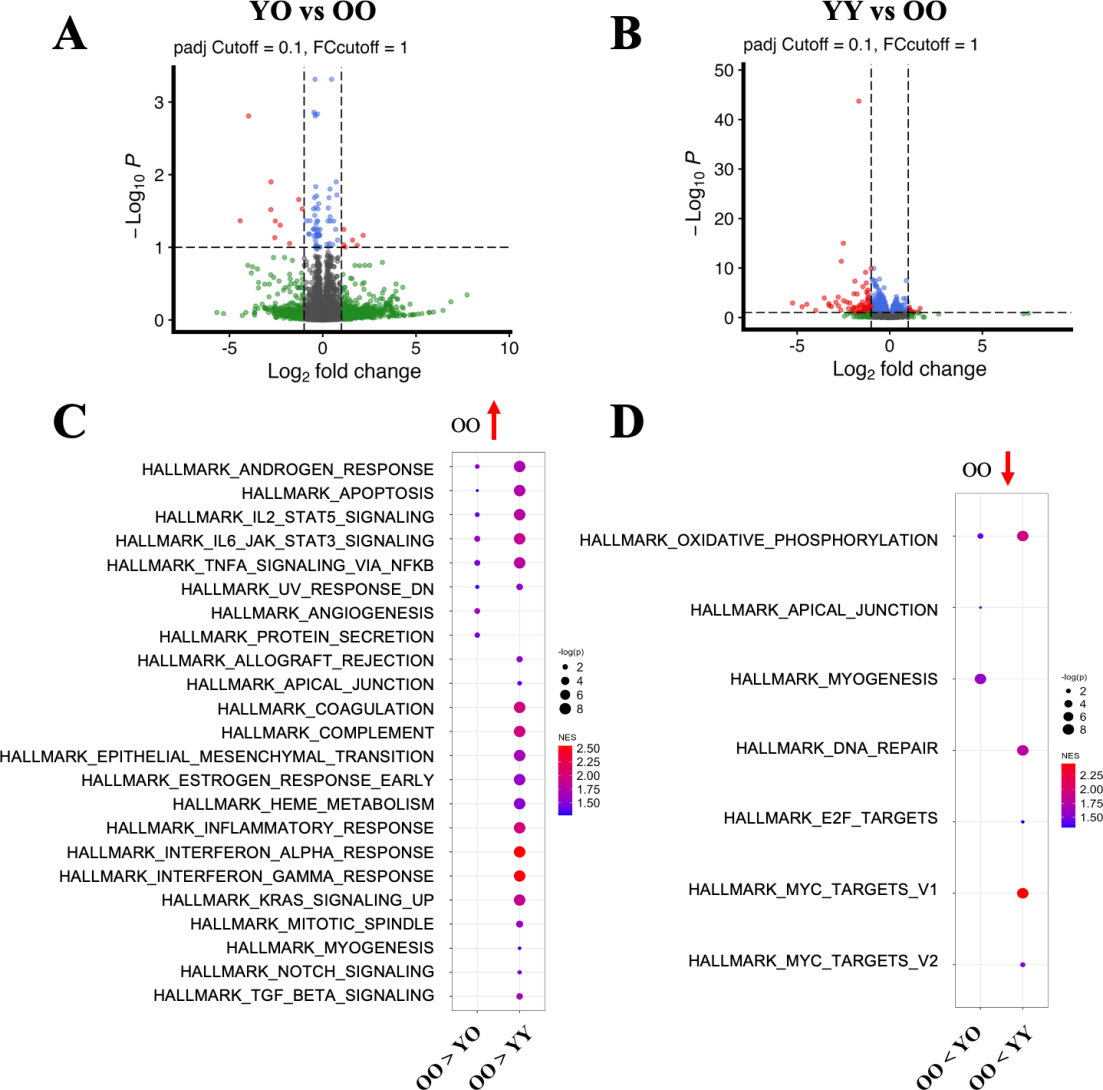
Transcriptomic differences of the amygdala in the OO and YO groups after FMT. (**A**) Volcano plot depicting differential gene expression in the OO vs YO comparison. Red dots on the top left and the top right represent upregulated genes (*P*adj < ene expression in OO vs YY comparison. Red dots on the top left and on the top right represent upregulated genes (*P*adj < 0.1 and fold changes > 1) in the OO group and YY group, respectively. (**C**) Dot plot depicting upregulated pathways in the OO group. Pathways involved in the OO vs YO comparison are listed in the left lane, and pathways involved in the OO vs YY comparison are listed in the right lane. All pathways listed are significant in each comparison (*P* < 0.05 and FDR < 0.25). (**D**) Dot plot depicting downregulated pathways in the OO group. Pathways involved in the OO vs YO comparison are listed in the left lane, and pathways involved in the OO vs YY comparison are listed in the right lane. All pathways listed are significant in each comparison (*P* < 0.05 and *P*adj < 0.25).

The amygdala is highly related to anxiety and depression phenotypes (29). To investigate how FMT with young microbiota impacts transcriptomic changes in the brains of the old recipients, we performed bulk RNA sequencing of the amygdala in the YY, OO, and YO groups. Differential gene analysis identified 17 and 92 genes that were significantly different between the YO and OO groups and between the YY and OO groups, respectively (Fig. 5A and B). To further identify enriched pathways between groups, we performed GSEA. We first investigated which hallmark pathways are upregulated in the OO group compared to the YO or YY group, that is, the pathways that are downregulated in the YO or YY groups. We found eight significantly upregulated pathways in the OO vs YO comparison and 21 upregulated pathways in the OO vs YY comparison (Fig. 5C). Among the eight significant pathways in the OO vs YO comparison, six overlapped with OO vs YY results, indicating FMT from young donors might partially rejuvenate the amygdala in old recipients. Some overlapping pathways such as apoptosis, IL6-JA-STAT3 signaling, and NFkB pathway are highly associated with inflammation and cellular senescence (30–33), suggesting that FMT with young fecal microbiota may alleviate inflammation and cellular senescence in old recipients’ amygdala. We next investigated which pathways were downregulated in the OO group compared to the YO or YY group, that is, the upregulated pathways in the YO or YY group. We found three significantly downregulated pathways in the OO vs YO comparison and five significantly downregulated pathways in the OO vs YY comparison (Fig. 5D). Oxidative phosphorylation was the only downregulated pathway identified in both the OO vs YY comparison and the OO vs YO comparison (Fig. 5D). These data suggest that FMT with young microbiota may upregulate mitochondrial oxidative phosphorylation, a well-known aging hallmark, in old recipients.

## DISCUSSION

Our study demonstrated that FMT with young microbiota has substantial positive effects on body composition, physical function, and psychological behaviors in the old recipients. These beneficial impacts were accompanied by changes in lipid and amino acid metabolism in the gut and serum, as well as transcriptomic changes in the amygdala region of the brain.

The percentage of body fat generally goes up with age (34), which is often associated with adverse health effects in aged populations (35). Unlike in humans, the percentage of lean mass in mice peaks during old age and remains relatively stable (36). In our study, FMT with young microbiota decreased the percentage of body fat and had no impact on the percentage of lean mass in old recipients. Changes in body fat were consistent with the stool and serum metabolites results, in which the YO group had a significant decrease of polyunsaturated acyl carnitine and saturated long-chain fatty acids, two metabolic biomarkers for aging (20, 37). Previous studies have shown that old microbiota has obesogenic characteristics (7). Our findings suggest that FMT with young microbiota may regulate lipid metabolism in the old recipient to prevent fat accumulation.

Numerous gut microbes have been implicated in weight control through the modulation of energy expenditure and storage (38). Our study found that *Turicibacter* was significantly enriched in the YO group across small intestines, large intestines, and stools. *Turicibacter* is commonly found in the gut of animals and humans, and its relative abundance is negatively correlated with a high-fat diet and host adiposity (39–41). *Turicibacter* administration decreases serum cholesterol and triglycerides in mice (42). Except for *Turicibacter*, *Lactobacillus* was also enriched in the YO group in small and large intestines but not in stools. *Lactobacillus* species are well-known probiotics and have been linked to multiple health benefits in human and animal studies. A recent systemic review reported that *Lactobacillus* administration reduces weight loss and improves metabolic status in overweight and obese individuals (43). *Lactobacillus* species suppress cellular senescence (44) and promote longevity and healthy aging (45–47). Thus, FMT with young microbiota may reduce body weight and percentage of fat via reconstruction of the gut microbiome of the old recipients. It also suggests that FMT with young microbiota may provide a novel solution to reduce body fat and improve aging-related metabolic conditions.

Some studies suggest that lipid profiles can predict physical frailty in old patients (48), and blood long-chain fatty acids were inversely correlated with longevity (49). The improved lipid metabolism in the YO group may contribute to improved frailty in the old recipient we observed. Circulating amino acid levels change with age, but the direction of changes varies with specific amino acids, sex, and macronutrient intakes (50–52). However, the consensus is that the anabolic effect of amino acids is attenuated in aged individuals compared to the young (53). The gut microbiome is involved in host amino acid metabolism (54), and probiotics such as *Lactobacillus* can enhance muscle strength (55). In our study, FMT with young microbiota increased serum amino acid levels such as pipecolate, an intermediate of lysine metabolism that is positively correlated with muscle mass and muscle strength (56). We found that FMT with young microbiota decreased aspartate and 3-hydroxyisobutyrate levels. The latter is a catabolic intermediate of the BCAA valine and has been identified as a strong marker of insulin resistance (57). Our study suggests that the FMT with young microbiota may improve the physical function of the old mice through the regulation of lipid and amino acid metabolism. However, the molecular pathways underlying young microbiota and host metabolism interactions need to be further elucidated in the future.

Aging is associated with an increased risk of depression and anxiety, and these psychological conditions, in turn, accelerate biological aging (58). The gut microbiome has been implicated in psychological behaviors via the gut–brain axis (59, 60). Previous studies have shown that FMT with young microbiota may affect neurological and psychological behaviors (12–14). However, findings on different behavioral tests from these studies are inconsistent. Our study using three behavior tests consistently showed that young microbiota improves depression and anxiety-like behaviors in the old recipients. Mechanisms involved in depression and anxiety are multifactorial. Characterization of lipidomic and amino acid changes in depression is of great interest. Sphingolipid and phospholipid metabolism play important roles in the development of anxiety and depression symptoms. An increase in lysophospholipids and sphingomyelin SM18:1 in blood has been associated with depression (61, 62), potentially through downregulation of systemic and neuroinflammation, which is a key pathophysiological change in individuals with depression. Our study showed that FMT with young microbiota decreased lysophospholipids such as 1-stearoyl-GPI (18:0) and multiple sphingolipids in the old recipients, suggesting the FMT-induced lipid changes may improve depression- and anxiety-like behavior. Neurotransmitters such as dopamine, gamma-aminobutyric acid (GABA), glutamate, norepinephrine, and serotonin have long been linked to depression. These neurotransmitters are either amino acids or are synthesized from amino acids. Studies have shown that L-tyrosine, methionine, and L-tryptophan in blood were decreased in patients with major depression (63). Interestingly, metabolic pathways involving these amino acids were upregulated in the serum of mice that received microbiota from young donors, suggesting a potential role of the gut microbiota in modulating amino acid metabolism and influencing mood-related pathways. One metabolite, catechol glucuronide, also has an anti-inflammatory effect (64). Aging is associated with reduced capacity of mitochondrial oxidative phosphorylation, which leads to impaired cellular metabolism (65). Although we did not measure mitochondrial function in the periphery, our RNAseq analysis in the amygdala suggested upregulation of oxidative phosphorylation from FMT with young microbiota. The improved mitochondrial function may in turn promote the utilization of fatty acids as energy, as we observed in the serum metabolome analysis. In addition, clinical and preclinical studies have suggested that inflammation is a key player in depression. We showed that FMT with young microbiota downregulated inflammatory pathways in the amygdala of the old recipients. Our study revealed multiple potential mechanisms, including host metabolism and neuroinflammation, that may contribute to observed behavior changes from FMT with young microbiota.

Consistent with a previous study (12), FMT with young and old microbiota to recipient mice did not completely recapitulate the microbiota in donor samples; rather, a new microbiome community structure was formed in the recipients after 2 months of FMT. This can be partly due to continuing changes in the microbiome within the study period, as all mice showed significant differences between baseline and the end of the study. Transplanted microbiota may alter existing microbial communities through direct competition or indirectly through modulation of host lipid and amino acid metabolism. Longitudinal mapping of the microbiome over the whole FMT process will help elucidate the microbiome community reconstruction process.

Our study has several limitations. First, only male mice were included in the current study since they exhibit a higher susceptibility to obesity and associated metabolic disorders. Female mice need to be included to examine the potential sex difference in the future. Second, antibiotic treatment before FMT may not completely deplete the gut microbiome, and the remaining microbiota may affect subsequent microbial colonization from FMT. Alternatively, germ-free mice may be considered for future studies. Third, the molecular pathways by which the young microbiota regulates lipid or amino acid metabolism remain unclear. Lastly, we acknowledge that the metabolic and transcriptomic changes following FMT do not establish causation for the behavioral changes observed in recipient mice, especially for complicated traits such as frailty and depression or anxiety. Future studies should focus on testing the causal connection between specific microbes, microbial and host metabolites, and physical function and behaviors.

In conclusion, our findings highlight the importance of FMT with young microbiota in improving body composition, physical function, and behaviors in old mice, which are linked to energy modulation in the periphery and transcriptomic changes in the brain. Our findings provide scientific evidence of targeting the gut microbiome to prevent and treat aging and age-related comorbidities in the future.

## Data Availability

16S rRNA gene data from the study can be accessed in the Sequence Reads Archive database with accession number PRJNA1048996.

## SUPPLEMENTAL MATERIAL

Supplemental figures msystems.01601-24-s0001.docx Fig. S1 and S2.

ASM does not own the copyrights to Supplemental Material that may be linked to, or accessed through, an article. The authors have granted ASM a non-exclusive, world-wide license to publish the Supplemental Material files. Please contact the corresponding author directly for reuse.
