# Supplementary material for: Young gut microbiota transplantation improves the metabolic health of old mice
Source: mSystems. 2025 May 30;10(6):e01601-24. doi: 10.1128/msystems.01601-24 (PMC12172422; doi:10.1128/msystems.01601-24)
Supplement: Supplemental figures — Fig. S1 and S2. [file msystems.01601-24-s0001.docx]

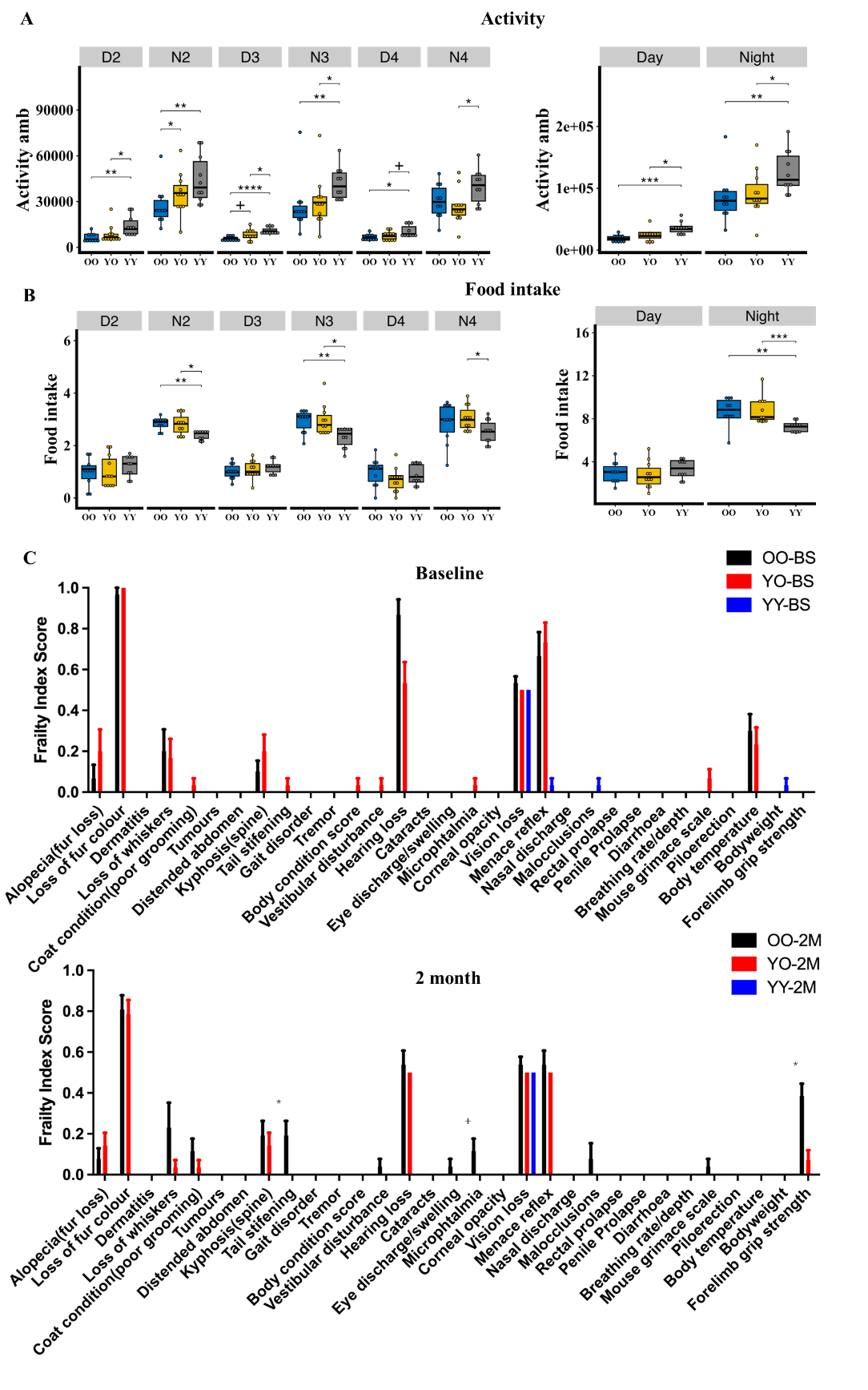


**Fig S1.** **Activity and food intake in OO, YO, and YY groups after two months of FMT.** (A) Activity levels, measured as the total number of ambulatory x-axis and y-axis infrared beam breaks, indicate that YO mice exhibited higher activity compared to the OO group during Night 2 and Day 3. In contrast, older mice demonstrated reduced activity relative to younger mice. Across all groups, activity levels were higher during nighttime compared to daytime. (B) Food intake analysis reveals that the YO and OO groups consumed similar amounts of food, both of which were greater than the intake observed in the YY group during nighttime. (C) The frailty index, comprising 31 individual measures, identified several significant differences between the OO and YO groups including tail stiffening, forelimb grip strength and microphtalmia. + p < 0.1, *p < 0.05, **p < 0.01, ***p < 0.001, ****p < 0.0001.


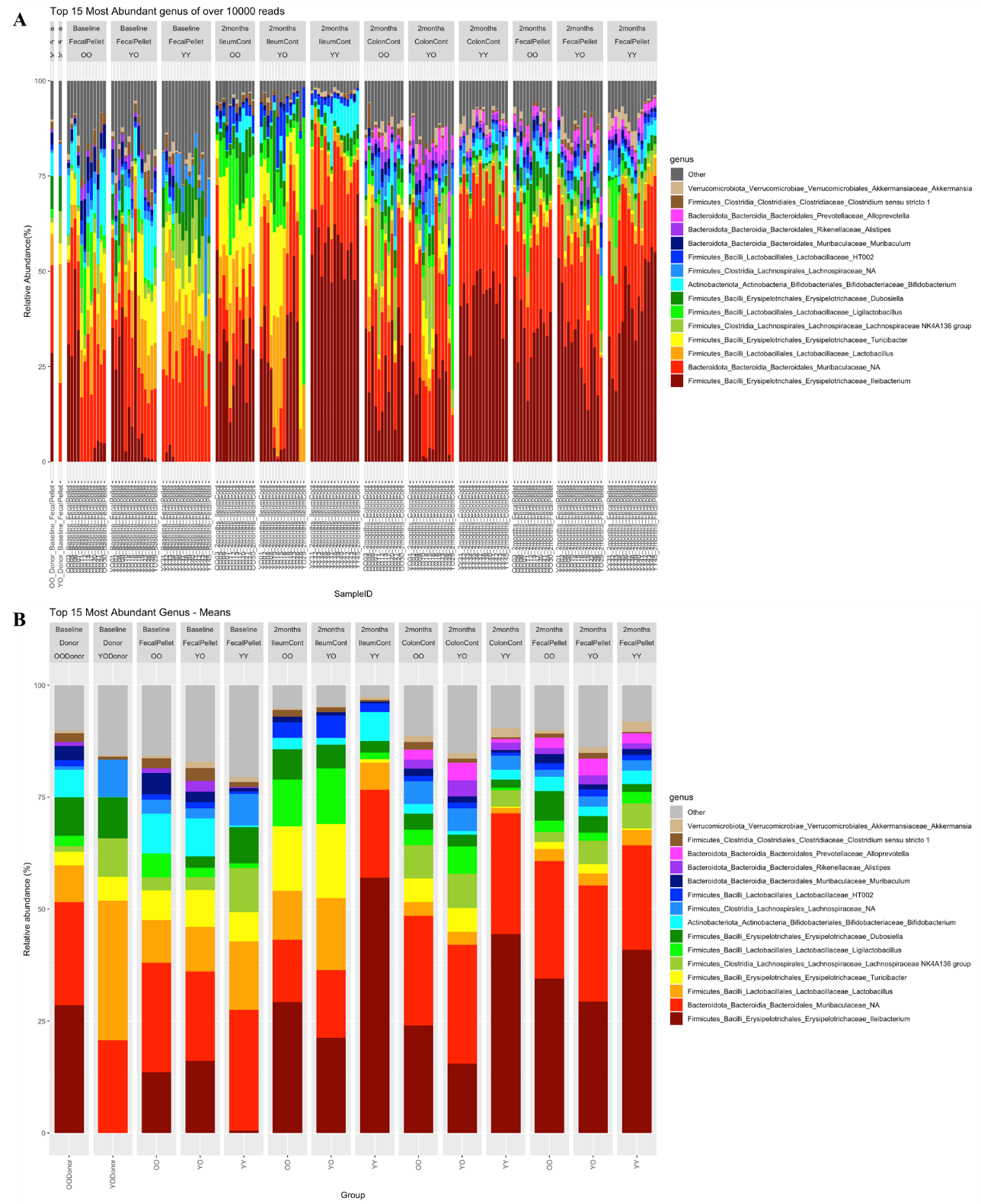


**Fig S2. Relative abundance of the top 15 genera in OO, YO, and YY groups before and after two months of FMT.** (A) Top 15 genera with over 10,000 reads in baseline fecal pellets, 2-month ileum content, 2-month colon content, and 2-month fecal pellets for OO, YO, and YY groups. (B) Average abundance of these top 15 genera across the same samples and groups.
